# Larger Gray Matter Volume in the Basal Ganglia of Heavy Cannabis Users Detected by Voxel-Based Morphometry and Subcortical Volumetric Analysis

**DOI:** 10.3389/fpsyt.2018.00175

**Published:** 2018-05-03

**Authors:** Ana Moreno-Alcázar, Begoña Gonzalvo, Erick J. Canales-Rodríguez, Laura Blanco, Diana Bachiller, Anna Romaguera, Gemma C. Monté-Rubio, Carlos Roncero, Peter J. McKenna, Edith Pomarol-Clotet

**Affiliations:** ^1^FIDMAG Germanes Hospitalàries Research Foundation, Barcelona, Spain; ^2^Centro de Investigación Biomédica en Red de Salud Mental, Madrid, Spain; ^3^Centre Fòrum Research Unit, Institut de Neuropsiquiatria i Addiccions, Parc de Salut Mar, Barcelona, Spain; ^4^IMIM (Hospital del Mar Medical Research Institute), Barcelona, Spain; ^5^Benito Menni Complex Assistencial en Salut Mental, Barcelona, Spain; ^6^Department of Radiology, Centre Hospitalier Universitaire Vaudois, Lausanne, Switzerland; ^7^Signal Processing Lab (LTS5), École Polytechnique Fédérale de Lausanne, Lausanne, Switzerland; ^8^Addictions and Dual Diagnosis Unit, Vall d'Hebron University Hospital-Public Health Agency of Barcelona (ASPB), Barcelona, Spain; ^9^Department of Psychiatry, Vall d'Hebron University Hospital, Barcelona, Spain; ^10^Psychiatric Service, University of Salamanca Health Care Complex, Salamanca, Spain; ^11^Institute of Biomedicine of Salamanca, University of Salamanca, Salamanca, Spain

**Keywords:** basal ganglia, cannabis, long-term users, MRI, voxel-based morphometry

## Abstract

**Background:** Structural imaging studies of cannabis users have found evidence of both cortical and subcortical volume reductions, especially in cannabinoid receptor-rich regions such as the hippocampus and amygdala. However, the findings have not been consistent. In the present study, we examined a sample of adult heavy cannabis users without other substance abuse to determine whether long-term use is associated with brain structural changes, especially in the subcortical regions.

**Method:** We compared the gray matter volume of 14 long-term, heavy cannabis users with non-using controls. To provide robust findings, we conducted two separate studies using two different MRI techniques. Each study used the same sample of cannabis users and a different control group, respectively. Both control groups were independent of each other. First, whole-brain voxel-based morphometry (VBM) was used to compare the cannabis users against 28 matched controls (HC1 group). Second, a volumetric analysis of subcortical regions was performed to assess differences between the cannabis users and a sample of 100 matched controls (HC2 group) obtained from a local database of healthy volunteers.

**Results:** The VBM study revealed that, compared to the control group HC1, the cannabis users did not show cortical differences nor smaller volume in any subcortical structure but showed a cluster (*p* < 0.001) of larger GM volume in the basal ganglia, involving the caudate, putamen, pallidum, and nucleus accumbens, bilaterally. The subcortical volumetric analysis revealed that, compared to the control group HC2, the cannabis users showed significantly larger volumes in the putamen (*p* = 0.001) and pallidum (*p* = 0.0015). Subtle trends, only significant at the uncorrected level, were also found in the caudate (*p* = 0.05) and nucleus accumbens (*p* = 0.047).

**Conclusions:** This study does not support previous findings of hippocampal and/or amygdala structural changes in long-term, heavy cannabis users. It does, however, provide evidence of basal ganglia volume increases.

## Introduction

Cannabis is the most commonly used illicit drug worldwide, consumed mainly by adolescents and young adults (1). Although a large number of people, especially cannabis users, consider cannabis to be a harmless drug, there is still debate about the possible behavioral and neurobiological consequences of cannabis use (2, 3).

Animal studies have consistently shown that Δ9-tetrahydrocannabinol (THC), the main psychoactive component of cannabis (4), induces neurotoxic changes in brain regions rich in CB1 cannabinoid receptors such as the hippocampus (5), amygdala (6), and cerebral cortex (4), affecting the processes associated with the maturational refinement of cortical neuronal networks (7, 8). A recent systematic review has shown that acute and chronic effects of cannabis impair human cognition, especially in the domains of verbal learning, attention, and memory (9), while the effects on other cognitive functions such as executive functions (10, 11) and IQ (12) remain unclear. However, use of this drug has been associated with social problems and a range of psychiatric disorders such anhedonia, anxiety, depression, and an increased risk of psychotic symptoms, especially in adolescent users (13–15).

In contrast to the animal literature, much less is known about the neurobiological consequences of cannabis use in the human brain. As a result of the growing concern about the use of this drug, several human studies using neuroimaging techniques were carried out, with inconsistent or inconclusive results. For example, some studies have not found regional or global gray matter (GM) abnormalities associated with cannabis use (16, 17) while others have shown volume reduction, especially in the amygdala, the hippocampus and parts of the prefrontal and the medial temporal cortex (3, 8, 18–24), or larger GM volume in the cerebellum (25). A study carried out by Lorenzetti et al. (19) analyzed other brain regions, using the same sample as Yücel et al. (18) such as the orbitofrontal cortex, the anterior cingulate cortex or the pituitary gland, and did not detect significant differences between cannabis users and healthy controls. On the contrary, Price et al. (26) identified lower GM volume in the orbitofrontal cortex, which was associated with attentional deficits. Similarly, Gilman et al. (27) assessed the nucleus accumbens and the amygdala using different types of neuroimaging analysis. The authors found a greater GM density in both left regions, and also showed a greater GM volume in the left nucleus accumbens. In contrast, these results were not replicated in another subsequent study (28), in which the authors used the same analysis techniques as those used by Gilman et al. (27) to evaluate the nucleus accumbens, amygdala, hippocampus, and cerebellum. Nevertheless, it should be mentioned that inclusion criteria regarding cannabis use in the healthy subjects of the study by Weiland et al. (28) required only the report of no cannabis use in the past 3 months. Therefore, these results cannot be extrapolated to long-term cannabis users.

In summary, taking into account the results of the last systematic review carried out by Nader et al. (29), the most important and replicated alterations in cannabis users have been identified in CB1 rich areas such as the hippocampus (3, 18, 23). Morphological changes in other brain regions are more controversial. Differences in the inclusion criteria allowed in the samples, and in particular those related to consumption, could be contributing to the mixed results. Another possible source of uncertainty is the use of different neuroimaging techniques, which may have different sensitivities to detect subtle morphological changes. Future studies using stricter and more appropriate criteria for collecting the sample, and employing different neuroimaging techniques to replicate results, will allow researchers to drawn more solid conclusions in this respect.

Based on previous structural findings, the present study aimed to further investigate the question of brain structural changes associated with cannabis consumption. We compared the GM volume of long-term, heavy cannabis users and matched controls. To provide more robust findings, two separate studies using two different MRI techniques were conducted. Each study used the same sample of cannabis users and a different control group, respectively. Both control groups were independent of each other. In the first study, whole-brain voxel-based morphometry (VBM) was used to compare the cannabis users against healthy controls. In the second study, a volumetric analysis of subcortical regions was performed to assess for differences between the cannabis users and a different sample of matched controls obtained from a local database of healthy subjects. We hypothesize that cannabis users will show neuroanatomical differences, especially in those brain regions rich in cannabinoids receptors and in regions implicated in substance abuse.

## Methods

### Participants

Fourteen long-term heavy cannabis adult users were recruited via three sources in Barcelona: (i) attenders at a specialist clinic in Vall de Hebron Hospital who wished to stop using the drug; (ii) inpatients in a unit in Benito Menni hospital specializing in drug-related disorders; (iii) a cannabis users association. They were questioned about lifetime drug use using the relevant section of the Structured Clinical Interview for DSM-IV-TR Axis I Disorders (SCID-I) (30) and also screened for psychopathology using the Computerized Diagnostic Interview Schedule IV (C DIS-IV) (31). The cannabis users were matched 1:2 with 28 non-drug-using controls (HC1 group) who were recruited via poster and web-based advertisement in the hospital and local community, plus word-of-mouth requests from staff in the research unit. They also were clinically interviewed to assess history of major mental illness.

Inclusion criteria were being 18–65 years of age, right handed and having an IQ score of >70. Cannabis users must have used cannabis at a minimum rate of 3 joints per day for at least 5 years and be willing to abstain from cannabis use for at least 24 h prior to assessment. Healthy controls were required to have not used cannabis at all during the last year, and underwent a screening interview to confirm that they did not have a lifetime history of more than sporadic use of this or other drugs. This last criterion was defined as a maximum consumption of a drug of 10 or more times in 1 year. Exclusion criteria were any history of neurological disorders or serious head injury; any history of mental illness and/or treatment with psychotropic medication; daily use of other drugs (cocaine, heroin, amphetamine, benzodiazepines, etc) or a lifetime history of alcohol abuse/dependency (defined according to DSM IV criteria), and MRI contraindications. In all cases, social alcohol use was permitted: following Spanish guidelines (32), the upper limit for this was set at ≤17 standard drink unit [(SDU); every SDU contains 10 g of pure alcohol] per week or 6 SDU in 24 h no more than once per month (men) and ≤11 SDU per week or 5 SDU in 24 h no more than once per month (women).

As well as being recruited to be similar in age and sex, the two groups were matched for estimated IQ using the Word Accentuation Test (Test de Acentuación de Palabras, TAP) (33, 34). This test is conceptually similar to the UK National Adult Reading Test (NART) (35) and the US Wide Range of Achievement Test (36) and requires pronunciation of Spanish words whose accents have been removed.

For the volumetric analysis of subcortical regions, the cannabis users were compared to a separate sample of 100 healthy matched adults collected from a local database of healthy subjects (HC2 group). They met the same inclusion and exclusion criteria as the HC1 in the VBM study.

All participants gave written informed consent. The research protocol (number PR-2012-03) was approved by the Clinical Research Ethics Committee of the Sisters Hospitallers (Comité de Ética de Investigación Clínica de las Hermanas Hospitalarias). All procedures were carried out according to the Declaration of Helsinki.

### MRI data acquisition

All participants underwent structural MRI scanning in the same 1.5 Tesla GE Signa scanner (General Electric Medical Systems, Milwaukee, Wisconsin, USA) located at the Sant Joan de Déu Hospital in Barcelona (Spain). High resolution structural T1 MRI data were acquired with the following acquisition parameters: matrix size 512 × 512; 180 contiguous axial slices; voxel resolution 0.47 × 0.47 × 1 mm^3^; echo (TE), repetition (TR), and inversion (TI) times 3.93, 2,000, and 710 ms respectively; flip angle 15°.

### Voxel-based morphometry

Structural data were analyzed with FSL-VBM, which implements an optimized voxel-based morphometry protocol (37) carried out with FSL tools (FMRIB Software Library, Analysis Group, FMRIB, Oxford, UK). FSL-VBM yields a measure of difference in local GM volume between populations. In a first step, structural images were brain-extracted using BET. Next, tissue-type segmentation was carried out and the resulting GM partial volume images were then aligned to Montreal Neurological Institute (MNI 152) standard space using the FSL tools FLIRT and FNIRT. The resulting images were averaged to create a study-specific template, to which the native GM images were then non-linearly re-registered. The registered partial volume images were then modulated to correct for local expansion or contraction. The modulated segmented images were then smoothed with an isotropic Gaussian kernel with a sigma of 4 mm.

Comparison of the cannabis users and the control group HC1 was carried out using a voxel-wise general linear model (GLM) and permutation-based non-parametric testing, correcting for multiple comparisons. These were made with the “randomize” tool implemented in FSL, using a cluster-based thresholding method with 10,000 iterations and initial cluster-forming threshold Z ≥ 3.0. Clusters were assessed for significance at *p* < 0.05, fully corrected for multiple comparisons across space.

As both groups were well matched for all demographic variables (for more details see the Results section) including age, sex, estimated IQ, as well as tobacco and alcohol consumption, the main analysis was performed without including covariates in the GLM. In order to investigate the effects of other consumption variables on the results, and to identify any potential correlation among these variables and GM volume, we conducted a separate complementary analysis including tobacco and alcohol consumption in the GLM. For completeness, we carried out an additional analysis by using the remaining variables, age, sex, and estimated IQ as covariates.

Anatomical locations of the significant clusters were determined with reference to the Harvard–Oxford cortical and subcortical structural atlases integrated into FSLView (part of FSL) and the AAL atlas (Anatomical Automatic Labeling), a macro-anatomical parcellation of the single subject MNI-space template brain within MRIcron software (http://people.cas.sc.edu/rorden/mricron/index.html).

### Subcortical volumetric analysis

The volume of subcortical structures was examined using FIRST, a model-based segmentation and registration tool part of FSL (38). This technique computes the volume of a number of predefined subcortical regions, including the caudate nucleus, putamen, pallidum, nucleus accumbens, hippocampus, amygdala, thalamus, and brainstem. The performance of FIRST has been shown to be comparable or better than other automated methods (38). The measurement of the nucleus accumbens and amygdala with 1.5T data has been considered to be difficult. As a further check, therefore, we verified that our means values in controls are very similar (i.e., plus/minus one standard deviation from the mean) to those reported in two independent studies using manual segmentation (39) and FIRST with 3.0T data (40).

The subcortical volumes were corrected for subject head size (i.e., intra-cranial volume) to obtain normalized volumes. This was achieved by multiplying the volume of each subcortical structure by the volumetric scaling factor resulting from the SIENAX tool (part of FSL). The normalized subcortical volumes of the cannabis users were compared with the volumes of matched adults collected from a local database of healthy subjects (HC2 group). In both groups, the structural MRI datasets were acquired on the same scanner and using the same MRI sequence. Moreover, the volumes were computed using the same FIRST tool.

Comparison of the cannabis users and the HC2 group was carried out using a GLM. The significance level was set at *p* < 0.05, corrected for multiple comparisons using Bonferroni correction. As in the main analysis of the previous VBM study, both groups were well matched for all demographic variables, including age, sex, and estimated IQ, hence, these were not included in the GLM. In this case, it was not possible to study the effect of tobacco and alcohol consumption due to lack of data in controls. Reported volumes were averaged across hemispheres. This approach was motivated by our VBM results, which produced bilateral and symmetrical significant differences (see Results section) and to reduce the total number of comparisons. Importantly, we explicitly checked that this approach did not mask any laterality effect by performing two different *t*-tests. Specifically, we verified in both cannabis users and controls that the mean volume of each structure is not different between hemispheres (i.e., all the *p*-values resulting from all tests were higher than *p* > 0.38). Moreover, for each individual structure, we verified that the inter-group differences between hemispheres are not different (i.e., all the *p*-values resulting from all tests were higher than *p* > 0.42).

Finally, a further analysis was conducted in cannabis users to examine if the volume measures correlate with the drug use measures: duration of cannabis use, age of onset and cannabis consumption per day. Pearson's linear correlation coefficients were calculated together with their *p*-values using a Student's distribution. The significance level was set at *p* < 0.05, corrected for multiple comparisons using Bonferroni correction.

## Results

### Demographic characteristics of the two groups

Sociodemographic and substance use characteristics of participants are shown in Table 1. It can be seen that the three groups used in both analyses were well matched for age, sex, race, and estimated IQ. Cannabis users and the control group HC1 did not show significant differences in tobacco and alcohol consumption, although there were differences in the number of smokers per group. However, the comparison with the control group HC2 could not be carried out due to lack of data. The cannabis users had used the drug heavily for fourteen years on average, with an average age of onset of seventeen.

**Table 1:** Sociodemographic and substance use characteristics of the samples.

	**CAN (*N* = 14) Mean (*SD*)**	**HC1 (*N* = 28) Mean (*SD*)**	**HC2 (*N* = 100) Mean (*SD*)**	**CAN vs. HC1 *p*-value**	**CAN vs. HC2 *p*-value**	**CAN vs. HC1 X^2^ Value**	**CAN vs. HC2 X^2^ Value**
Age (years)	30.14 (5.21)	31.29 (6.58)	31.31 (6.93)	0.57	0.56		
Gender (male/female)	4/10	8/20	40/60			1.00	0.41
Race (Caucasian/others)	14/0	28/0	100/0			.	.
Estimated IQ (TAP)	101.08 (7.14)	104.04 (5.46)	103.43 (8.18)	0.15	0.32		
Duration of cannabis use (years)	14.36 (6.69)	–	–				
Age of onset	17.07 (2.92)	–	–				
Cannabis consumption (joints/day)	8.36 (3.81)	–	–				
Tobacco consumption (cigarettes/day)	5.58 (6.52)	1.74 (3.80)		0.08			
Tobacco consumption (n° subjects)	8	5				<0.01	
Alcohol consumption/week (drink)	1.08 (1.31)	0.83 (0.83)		0.58			
Alcohol consumption (n° subjects)	6	8				0.35	

*CAN, Cannabis users; HC1, Healthy controls 1 analysis; HC2, Healthy controls 2 analysis, SD, standard deviation; n°, number*.

### VBM comparison of cannabis users and controls

**Figure 1:**
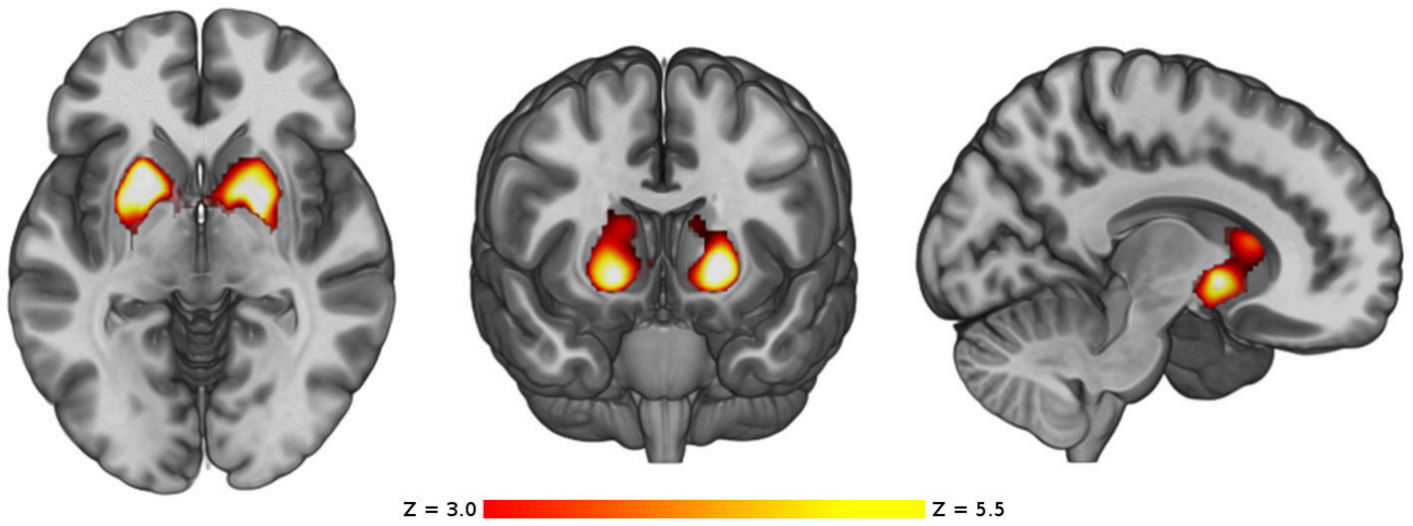
VBM comparison between the cannabis users and the control group HC1. Regions showing significant larger volume in the cannabis users are shown in red-yellow. The color-bar shows the z-score scale. The right side of the images represents the right side of the brain.

**Figure 2:**
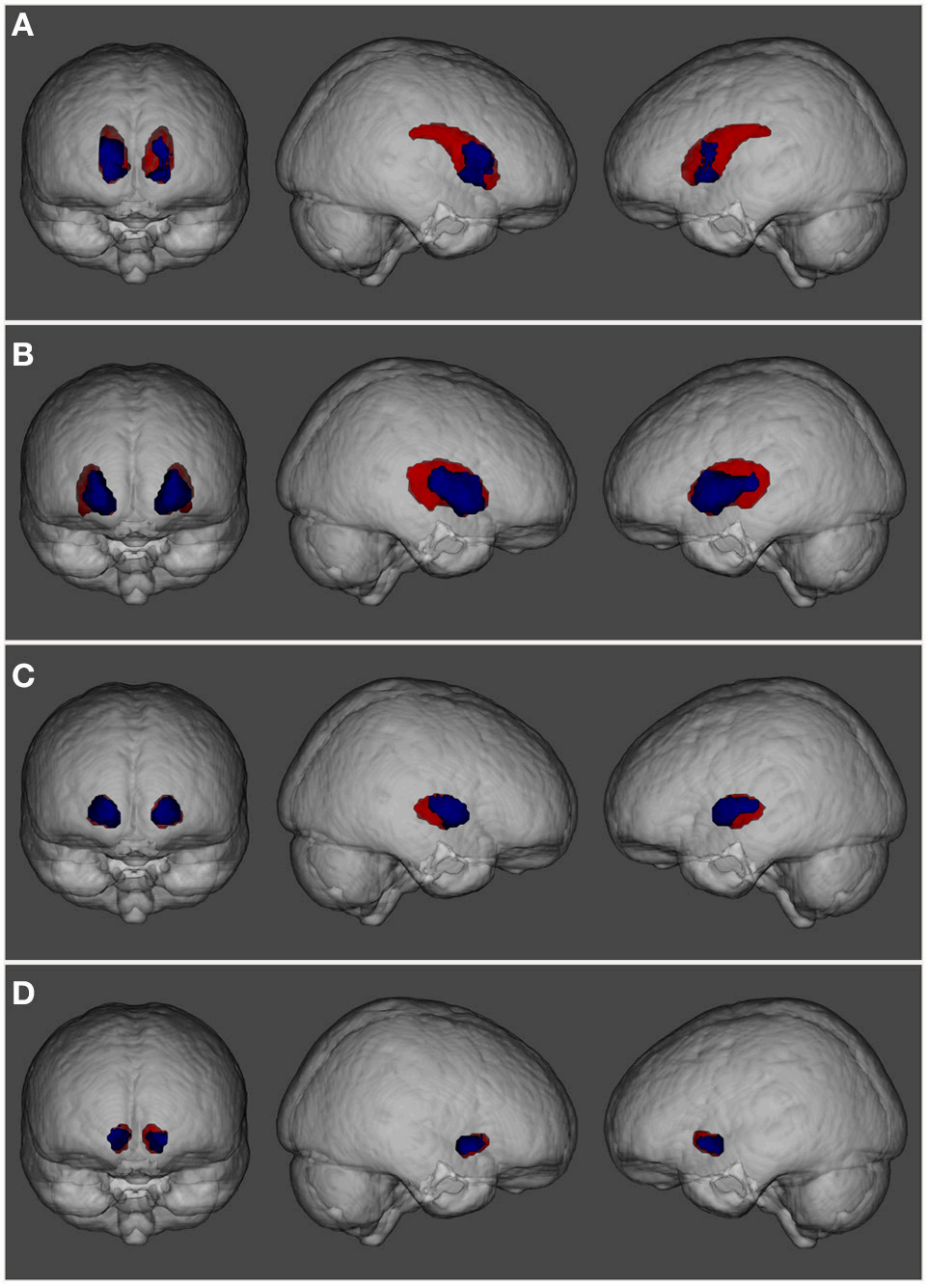
Three-dimensional view showing the spatial localization of volume abnormalities in different regions of the basal ganglia: **(A)** caudate nucleus, **(B)** putamen, **(C)** pallidum, and **(D)** nucleus accumbens. Basal ganglia structures are depicted in red while regions showing volume alterations are shown in blue.

The main analysis without using covariates revealed that, compared to the control group HC1, the cannabis users did not show cortical differences nor smaller volume in any subcortical structures but showed a cluster of larger GM volume in the basal ganglia [*p* < 0.001, three main peaks in MNI standard space: peak1 (−18, 8, −2), z-score = 5.53; peak2 (24, −8, 8), z-score = 4.33; peak3 (12, 18, 10), z-score = 4.05; see Figure 1]. This cluster included parts of the caudate, putamen, pallidum and nucleus accumbens, bilaterally. In order to better visualize the spatial location and overlapping within these structures, a three-dimensional view of these subcortical regions is depicted in Figure 2.

The complementary analysis using tobacco and alcohol consumption as covariates in the GLM revealed a very similar pattern of inter-group differences, affecting the same subcortical structures in both hemispheres. In particular, the cannabis users showed two bilateral clusters of larger GM volume [cluster1 on left hemisphere, *p* = 0.016, main peak in MNI standard space (−18, 8, −2), z-score = 5.28; cluster2 on right hemisphere, *p* = 0.013, main peak in MNI standard space (22, 2, −2), z-score = 5.03). The analysis did not find any cluster with significant positive or negative correlation between GM volume and tobacco or alcohol consumption. Similarly, the complementary analysis using age, sex, and IQ as covariates revealed two bilateral clusters of larger GM volume [cluster1 on left hemisphere, *p* = 0.02, main peak in MNI standard space (−18, 8, −2), z-score = 5.09; cluster2 on right hemisphere, *p* = 0.016, main peak in MNI standard space (20, 16, −10), z-score = 5.03]. The analysis did not reveal any significant positive or negative correlation between the covariates and GM volume.

### Comparison of subcortical volumes in the cannabis users against a database of healthy subjects

**Figure 3:**
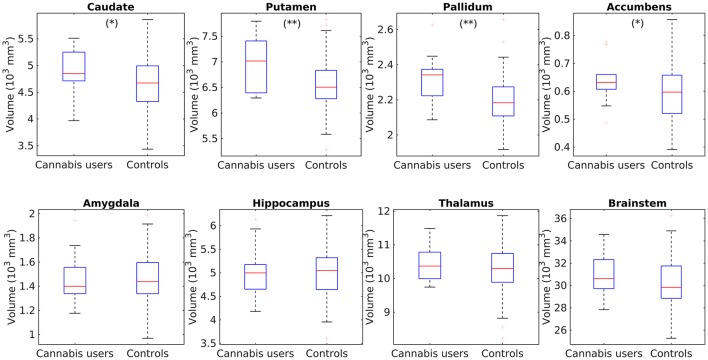
Box plots showing the comparisons of subcortical volume in the caudate, putamen, pallidum, accumbens, hippocampus, amygdala, thalamus, and brainstem between cannabis users and the control group HC2. ^**^Individual tests significant at a corrected *p* < 0.05, ^*^Significant only at the uncorrected level *p* < 0.05.

After correcting for multiple comparisons, the analysis did not reveal any subcortical structure with larger volume in the HC2 group. In contrast, the cannabis users depicted significant larger volumes in the putamen (*p* = 0.001) and pallidum (*p* = 0.0015). Interesting, subtle trends, only significant at the uncorrected level, were also found in the caudate (*p* = 0.05) and nucleus accumbens (*p* = 0.047). The findings are shown in Figure 3.

The correlation analysis in the cannabis users did not show any significant positive or negative correlation between the subcortical volumes and the duration of cannabis use, the age of onset, and the cannabis consumption per day.

For completeness of this report, in Table 2 we provide information about the mean values and standard deviation of all the evaluated structures in both populations.

**Table 2:** Volume of subcortical structures.

**Structures (mm^3^)**	**HC2 (*N* = 100)**	**CAN (*N* = 14)**
	**Mean (SD)**	**Mean (SD)**
Caudate	4,686.3 (454.2)	4,906.4 (393)
Putamen	6,549.5 (454.4)	6,996.5 (539.8)
Pallidum	2,190.4 (136.9)	2317 (131)
Accumbens	593.7 (99.9)	642.5 (83.7)
Amygdala	1,462.1 (205.9)	1,447.4 (210.6)
Hippocampus	4997.9 (478.7)	5,025.3 (571.1)
Thalamus	10,278.1 (645.2)	10,424.3 (491.6)
Brainstem	30,169.1 (2102)	31,024 (1985.1)

*Reported volumes were averaged across hemispheres and corrected for subject head size (i.e., intra-cranial volume). This was done by multiplying the volume of each subcortical structure by the volumetric scaling factor resulting from the SIENAX tool (part of FSL). This operation transforms the volumes from the native space to the average MNI space. CAN, Cannabis users; HC2, Healthy controls 2; SD, standard deviation*.

## Discussion

The aim of this study was to evaluate whether there are structural brain differences between heavy cannabis users and healthy subjects, particularly in brain regions rich in cannabinoids receptors and in regions implicated in substance abuse A modulated VBM analysis showed that cannabis users did not present cortical regional differences nor smaller volume of GM in any subcortical structures in comparison with the healthy controls (HC1) group. However, the analysis showed a significant cluster of larger GM volume in the basal ganglia, involving the caudate, putamen, pallidum and nucleus accumbens, bilaterally. The first complementary analysis did not reveal any significant positive or negative correlation between tobacco and alcohol consumption and the GM volume. However, the lack of results in this analysis were to be expected due to the subjects' low levels of consumption. The second complementary correlation analysis also showed no difference between age, sex, IQ, and the GM volume. Therefore, these variables also had no influence on the main results found in this study. The VBM findings were also supported by a comparison of the volumes of a range of anatomically defined subcortical structures between the cannabis users against a large database of healthy subjects (HC2 group), which also showed larger volumes for the putamen and pallidum nuclei. The caudate and accumbens were not statistically significant after correcting for multiple comparisons, but we observed a trend at the uncorrected level (*p* < 0.05). These volumetric changes did not correlate with any drug use measure.

Our finding of larger basal ganglia volume is similar to that of Gilman et al. (27), who compared 20 recreational adolescent users and 20 non-users using VBM and found a subcortical cluster of increased volume involving the left nucleus accumbens/putamen and amygdala. The increase in the left accumbens, but not that in the amygdala, was also found in a subsequent ROI analysis and was correlated with frequency of drug use. These results are consistent with animal studies which showed an increase in the number of dendritic branches and an increase in density of dendritic spines in the nucleus accumbens in amphetamine, cocaine, nicotine and cannabis treated rats (27). In addition, a recent meta-analysis of functional neuroimaging studies carried out by Yanes et al. (41) observed an increase in activation within the striatum among cannabis users compared to the controls in cognitive, social cognitive, affective, perceptual, and/or motor tasks. Interestingly, increased basal ganglia volume, as we found in cannabis users, has also been reported in association with long-term use of another drug, cocaine. Ersche et al. (42) compared 60 chronic cocaine-dependent individuals with 60 healthy controls using VBM, and found evidence of widespread cortical GM reductions, but also a significant increase in subcortical GM volume, which affected basal ganglia structures including the putamen, caudate nucleus and pallidum, and also the cerebellum. Cocaine, like other stimulants such as methamphetamine, exerts an important both acute and chronic neurotoxic changes in dopaminergic neurons, and potentially its use could be responsible for the basal ganglia changes by inducing neuroinflammatory processes. In this way, for example, two volumetric studies documented that active methamphetamine users showed an increase in the basal ganglia, including the caudate nucleus, globus pallidus, and putamen (43, 44), while another study showed that those users who were abstinent for more than 20 months presented normal volumes in these cerebral regions, but a minor alteration in the shape of the corpus callosum (45). These results suggest that the use of methamphetamine could induce inflammation or reactive gliosis, which may normalize with longer abstinence (46). Taking into account our results, the same neurobiological process may be occurring in chronic cannabis users. In fact, one study carried out in adolescent rats showed that THC exposition induces a persistent neuroinflammatory state in the prefrontal cortex, characterized by increased levels of the inflammatory mediators, TNF-α, iNOS, and COX-2, and reduction of the anti-inflammatory cytokine, IL-10 (47). However, further research, in both animal and humans, is needed to confirm this hypothesis and to better understand the neurobiological effects of cannabis.

On the contrary, our data do not support the results of previous studies which found smaller right ventral striatum volumes (~3.5%) in cannabis users (48), or found no significant differences between users and healthy controls (21, 28). On the other hand, our findings are notably different from those who reported reduced hippocampal (3, 18, 20, 22, 23, 49) and amygdala volume (18, 48), as well as a smaller volume in cortical areas (24, 26). However, as noted in the introduction, other studies have had negative findings for these structures (21, 23), including one in which the participants had heavy use for at least 2 years (25) and another in which they had taken the drug more than 5,000 times (17). A recent large study by Pagliaccio et al. (48) suggests a possible reason for these discrepant findings: they examined the influence of both hereditary factors and cannabis use on the volumes of the whole brain, hippocampus, amygdala, ventral striatum, and orbitofrontal cortex in a sample of 483 individuals made up of 3–4 siblings, and in some cases twin pairs. The left amygdala and right ventral striatum were found to be smaller (by 2.3 and 3.5%, respectively in those with a history of cannabis users), but the association between left amygdala volume and cannabis use emerged as being largely a function of shared genetic factors (whether this also applied to the smaller right ventral striatum volume found in cannabis users was unclear). These results are of great interest because they could suggest that brain alterations found in cannabis users could be explained by genetic factors and not exclusively by drug use. However, further research is needed to confirm this hypothesis.

The main limitation of this study is the relatively small sample size. However, with the subcortical volumetric analysis, we replicated the findings from the VBM analysis. The fact that drug users frequently take multiple drugs, and often alcohol as well, probably means that for the foreseeable future findings about the consequences on the brain of long-term heavy cannabis use will have to rely on mega-analysis or meta-analysis using data from a number of relatively small studies. Although all healthy controls were clinically interviewed and have never had a history of more than sporadic use of cannabis or other drugs, data on substance abuse in the past were not systematically collected for the majority of the 100 healthy controls of the second analysis (HC2 group) (especially tobacco and standard alcohol units consumption). This lack of information has not allowed us to study any potential effect related to other consumption variables. However, in the first study (HC1 group), we did not find any correlation between these variables and GM volume.

To conclude, despite the small sample of our VBM study, the results partially support our hypothesis, which suggested that long-term cannabis use may be associated with changes in critical brain regions implicated in substance abuse such as the basal ganglia. Further research with larger samples and with multimodal analysis techniques are needed to confirm these results.

## Author contributions

PM and EP-C have participated in the conception and design of the study. AM-A and BG wrote the first draft of the manuscript, with supervision from PM and EP-C. EC-R and GM conducted the analysis of images. LB, AR, DB, and CR participated in the interpretation of the data. All authors contributed to the revisions of the manuscript and all have approved the final version.

### Conflict of interest statement

The authors declare that the research was conducted in the absence of any commercial or financial relationships that could be construed as a potential conflict of interest.
